# How do citizens with low health insurance literacy choose a health insurance policy in the Netherlands? An interview study

**DOI:** 10.1186/s12913-024-12062-0

**Published:** 2024-12-18

**Authors:** Laurens Holst, Anne Brabers, Jany Rademakers, Judith de Jong

**Affiliations:** 1 Nivel, the Netherlands Institute for Health Services Research, PO Box 1568, Utrecht, 3500 BN the Netherlands; 2 CAPHRI, Maastricht University, PO Box 616, Maastricht, 6200 MD the Netherlands

**Keywords:** Health insurance literacy, Health insurance policy, Netherlands

## Abstract

Various countries have implemented a choice-based health insurance system. For such systems to function as intended, it is crucial that all citizens have the opportunity to make well-informed decisions with regard to their health insurance policy. There is, however, ample research evidence to suggest that many citizens may lack the required skills to do so, thus increasing the likelihood of suboptimal insurance choices and incurring unexpected costs. The current study explored what barriers citizens in the Netherlands, with low health insurance literacy (HIL), face when selecting a health insurance policy, and what their specific needs and preferences are regarding information and support. Semi-structured interviews (online or telephone) were performed between January 2023 and April 2023 among sixteen with a low level of HIL. The data were analyzed using thematic analysis methods. Our results show that not all citizens with low HIL are sufficiently motivated to actively look for alternative health insurance options every year. This is partly due to the feeling that it is a task imposed upon them by the government, and because choosing a health insurance policy is perceived as too complicated and not sufficiently worthwhile. There is a need among citizens with low HIL for clear and practical information about health insurance policies, especially from the government and health insurers. In addition to this need, we recommend enhancing awareness regarding the consequences of neglecting to explore such health insurance options. Moreover, more personal attention for the process of selecting a health insurance policy should be offered to vulnerable groups such as those who possess insufficient digital skills.

**Supplementary Information:**

The online version contains supplementary material available at 10.1186/s12913-024-12062-0.

## Introduction

Various countries, such as the United States, Switzerland, and the Netherlands, have implemented a choice-based health insurance system [1–3]. Within these systems, citizens have the opportunity to select a health insurance policy with certain conditions from a wide range of different policies from multiple health insurers. In theory, such systems have two main advantages. Firstly, it empowers citizens to select a health insurance policy that aligns with their needs and preferences [4]. Secondly, it should incentivize insurers to maintain high standards of care, service quality, and competitive pricing, driven by the prospect that their current insured could switch to another health insurer [2, 5–7].

For choice-based health insurance systems to function as intended, it is crucial that all citizens have the opportunity to make well-informed decisions with regard to their health insurance policy [8]. There is, however, ample research evidence demonstrating that this is not the case in practice. Besides the fact that some citizens are simply not interested in delving into the details of health insurance policies and their various options [9–11], the task of selecting a suitable health insurance policy appears overly challenging for a substantial portion of citizens. Many citizens lack the required skills to make both informed decisions when selecting a health insurance policy, and also to use it effectively once enrolled [12–14]. Consequently, these citizens may find themselves sub-optimally insured, which may lead to problems with access to the healthcare they desire or require [13, 15–17]. Additionally, they may encounter unexpected costs when they anticipate reimbursements for certain expenses for which they are not insured.

In order to make well-informed decisions related to health in general, different types of skills are required. Three dimensions can be distinguished: (1) cognitive attributes (knowledge, functional health-related skills, comprehension and understanding, appraisal and evaluation, critical thinking); (2) behavioural and operational attributes (seeking and accessing information, communication and interaction, application of information, other context-specific skills, citizenship); and, (3) affective and conative attributes (self-awareness and self-reflection, self-control and self-regulation, self-efficacy, interest and motivation) [18]. The specific skills that people need when selecting a health insurance policy are integrated into the concept of health insurance literacy (HIL) which can be defined as ‘*the extent to which consumers can make informed purchase and use decisions regarding health insurances’* ([19] p.3). HIL is connected to the concept of health literacy, which refers to individuals’ competencies in accessing, comprehending, evaluating, and effectively applying, health-related information [20]. However, HIL stands apart due to its focus on the benefits of health insurance and its cost-sharing responsibilities [21]. In this study, we focus on the skills of citizens with low HIL in selecting and making use of a health insurance policy.

A recent study which concentrated on the choice-based health insurance system in the Netherlands, showed that there is a relationship between the level of HIL among citizens and their perceptions of the health insurance selection process [22]. Citizens with lower HIL levels perceive the process of choosing a health insurance policy more often as difficult, uninteresting, and boring. They will also attach less importance and value to it. In addition, they make less frequent use of their option to switch to another health insurer than citizens with higher HIL levels [22].

Since the introduction of the Healthcare Insurance Act in 2006, citizens in the Netherlands are obliged to have a basic health insurance policy and can choose from a variety of possible private insurers each year [4]. For instance, in 2023, citizens had the choice of selecting from 60 different basic policies offered by 20 insurers [23]. Additionally, citizens can also opt for a voluntary deductible or a supplementary health insurance policy [4]. The voluntary deductible is an optional increase in the amount that citizens must pay out of their own pocket before becoming eligible for insurer reimbursements. In return, they receive a reduction in their premium costs. Supplementary health insurance policies are voluntary ones that offer coverage for additional healthcare services such as dental care and physiotherapy.

For choice-based health insurance systems to function as intended, it is crucial that all citizen have the opportunity to make well-informed decisions regarding their health insurance. To this aim, the current study intends to gain a better understanding of how citizens with a low level of HIL choose a health insurance policy. We want to explore in greater depth what barriers they face during the policy selection process, and what their specific needs and preferences are regarding information and support. The following research questions are addressed in this study:

- To what extent do citizens with low HIL find choosing a health insurance policy important, interesting, and difficult? And why?
- What steps do citizens with low HIL take when choosing a health insurance policy?
- What barriers do citizens with low HIL experience when choosing a health insurance policy?
- How, and by whom, do citizens with low HIL want to be supported when choosing a health insurance policy?

## Method

### Study design and participants

Data were collected by conducting in-depth and semi-structured interviews. All the participants of the current study were recruited from the Nivel Dutch Health Care Consumer Panel (DHCCP). The aim of this panel is to measure, at a national level, opinions on, and knowledge about, healthcare and the expectations and experiences with healthcare [24]. The DHCCP is an access panel which consists of a large number of individuals who have voluntarily committed themselves to responding to healthcare-related questions regularly. On becoming a member of the DHCCP, participants are informed of the purpose, scope, method, and use of the panel. Based on this information, they can then choose whether or not to participate in the panel. Written or digital informed consent is obtained upon the registration of a new panel member. According to Dutch legislation, approval by a medical ethics committee is not required for conducting research through the panel [25].

**Fig. 1:**
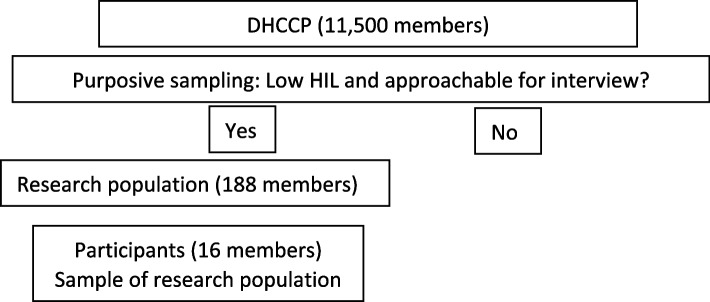
Selection of the participants

At the time of this study, the panel consisted of approximately 11,500 members of whom various demographic characteristics were known, such as age, gender and HIL-level. Purposive sampling was conducted (Fig. 1) to ensure all the panel members approached were, firstly, classified as citizens with low HIL (a HIL score lower than 60) using the HILM-NL which is a reliable instrument for measuring health insurance literacy among citizens in the Netherlands [26, 27]. Secondly, they must have indicated that they could be approached for an interview during the panel registration. A sample of 188 members was drawn from the DHCCP based on these two criteria. Within this sample, sixteen interviews were scheduled by telephone, with an effort to include a diverse sample of panel members with regards to age and gender. Prior to the interview, all participants were given verbal and written information about the aim and scope of the interview, and all participants gave verbal informed consent at the start of the interview. Data were pseudonymized, analyzed, and processed in accordance with the privacy policy of the Dutch Health Care Consumer Panel. The panel complies with the General Data Protection Regulation (GDPR).

### Data collection

Sixteen in-depth and semi-structured interviews were performed. All the interviews were conducted by the first author (LH) between January 2023 and April 2023. There were no personal or professional relationships between the participants and the interviewer. The interviews were conducted using a topic list, which consisted of open-ended questions to gain in-depth perspectives. The topic list was developed collaboratively by three of the four authors (LH, AB, JdJ). In line with the research questions, the topic list consisted of four main topics: (1) the degree of importance, interest, and difficulty in choosing a health insurance policy; (2) the perceived barriers when choosing a policy; (3) the steps taken while choosing a policy; (4) the desired support when choosing a policy. An overview of the topic list is presented in Supplementary File 1. The duration of the interviews was approximately 60 min. The interviews were conducted online (Microsoft Teams) or by telephone, depending on the preference of the participant. All interviews were audiotaped with permission of the participants.

### Data analysis

Following each interview, the most important findings were documented and carried forward to the next. This was part of an iterative process of data collection, namely data analysis – new data collection. After eight interviews, the initial findings were discussed with three of the four authors (LH, AB, JdJ), and points of interest were identified for the remaining eight interviews. After sixteen interviews, data saturation was reached, and in consultation with the co-authors, it was decided not to schedule additional interviews.

After all interviews, the audiotapes were transcribed verbatim and anonymized. Braun and Clarke’s six-step method for inductive thematic data analysis was used to derive themes from the data [28, 29]. To enhance the trustworthiness of the study, researcher triangulation was applied [30]. Two researchers (LH, AB) searched for codes independently, focused on two of the four research questions. After this the codes were discussed. The transcripts of the remaining two research questions were coded by one researcher (LH) and randomly checked by the other researcher (AB). During the first step of the coding process, the researchers thoroughly read the complete interview transcripts to familiarize themselves with the data. Secondly, the researchers created initial codes to capture potentially relevant data (open coding). Discrepancies between the researchers regarding these codes were discussed until a consensus was reached. In the third step, the transcripts were examined systematically in order to identify overarching themes, forming the foundation for the coding tree (axial coding). Fourthly, a review of the themes was conducted in relation to the data (selective coding), and the coding tree was assessed within the entire research team to enhance its overall trustworthiness [30]. During the fifth step, distinctive descriptions were formulated for each theme. Finally, the themes and distinctive descriptions were compiled to provide a comprehensive overview of the findings. In addition, we used a ‘peer debriefing’ strategy to further strengthen the trustworthiness of the study [30]. This involved discussing a draft of this paper, including the “Results” sections, during an academic meeting with a group of peer researchers who were not involved in the study. Following this peer debriefing, minor adjustments were implemented in the draft paper aimed particularly at clarifying the context of the findings. The coding process was supported by the MAXQDA program (Release 22.1.1). For writing this article, we used the Consolidated criteria for Reporting Qualitative research (COREQ) checklist [31].

## Results

### Participants

All sixteen panel members completed the interview. Table 1 provides an overview of their characteristics.

**Table 1:** Characteristics of the participants

		n
Total		16
Gender	Male	8
	Female	8
Age (Range)		39–80
Region	North	7
	Middle	7
	South	2
Highest completed education level^a^	Low	-
	Intermediate	11
	High	5
Household net income per month in euros	< 1.750	4
	1.750–2.700	5
	> 2.700	7
Self-reported health	Bad / fair	5
	Good	8
	Very good / excellent	3

^a^Low = none, primary school or pre-vocational education. Intermediate = secondary or vocational education. High = professional higher or university

### Interview results

**Fig. 2:**
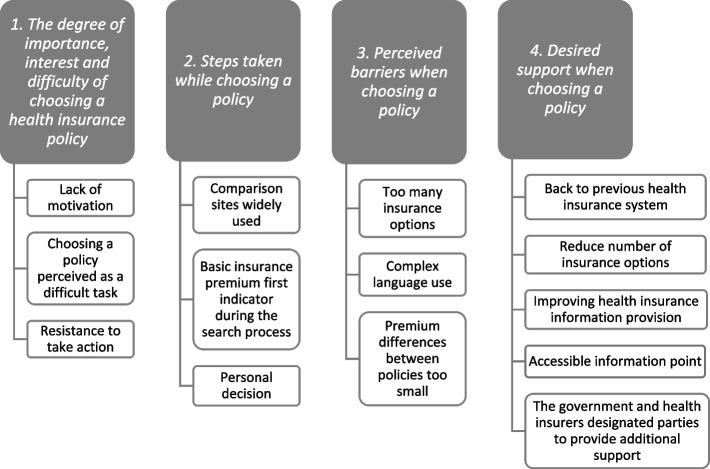
Overview of the results

The interview results are described below for each research question. Figure 2 provides an overview of the results.

1. *The degree of importance*,* interest*,* and difficulty in choosing a health insurance policy*

The participants in this study generally emphasized that choosing a health insurance policy is important. However, they expressed a lack of motivation to explore, actively, their various health insurance options. They did not find the topic interesting enough, with several participants stating a reluctance to engage with it on an annual basis. The process of identifying the differences between policies is too time-consuming, according to the participants.

*‘I understand its importance*,* but I do not find it interesting. I lack the patience for it. It seems too time-consuming*,* and*,* frankly*,* I consider it a waste of time. That’s how I see it’. (Participant #3)*

A few participants also indicated that they did not want to look into alternative health insurance policies, because they were satisfied with their current one.

In addition, most participants pointed out that choosing a suitable health insurance policy is a difficult task. In particular, comparing the advantages and disadvantages of health insurance policies is considered hard.

*‘Sorting through it all proves challenging for me. What distinguishes one from the other? Trying to compare across all those insurers is indeed a complex task*,* given their subtle differences. I’ve tried it before*,* but I think everything ended up being the same’. (Participant #15)*

Furthermore, for the majority of the participants, the obligation to choose a health insurance policy feels like a mandate imposed by the government following the introduction of the Healthcare Insurance Act in 2006. Before the introduction of this Act, many citizens were automatically enrolled in a national health insurance fund. Several participants expressed a sense of resistance when it comes to taking action in choosing a health insurance policy.

*‘I feel like something is being imposed on me that was previously taken care of by the national health insurance fund*,* and now I have to invest a significant amount of time and thought into it. In that sense*,* I don’t find it interesting because I am essentially compelled to engage in something I never had to do before’. (Participant #11)*

2. Steps taken while choosing a policy

All participants stated that they were well-informed about the yearly window during which they could switch to another health insurance policy. The participants that search for another policy indicated that when they do look into their health insurance options, they often use comparison sites to gather information about health insurance policies, and, to a lesser extent, they visit the health insurers’ websites or, for example, the website of the Dutch Consumers Association ‘Consumentenbond’. Typically, they first assess the premium for the basic health insurance policy, followed by a closer examination of the terms and conditions. They also examine closely the supplementary policies. Some participants indicated that they once, or frequently, make contact with the health insurer by telephone with questions throughout the search process. For most participants, choosing an insurer and policy is perceived as a personal decision, predominantly made independently or within the family. Discussions with friends about this decision occur only occasionally.

*‘Well*,* the decision about health insurance is a personal matter*,* and what works for one person may not necessarily be suitable for another. It is not a one size fits all situation where you can simply replicate someone else’s choice. It doesn’t work like that’. (Participant #4)*

3. Perceived barriers when choosing a policy

Most participants indicated the overwhelming number of options when choosing a health insurance policy. It is, according to them, difficult to get a clear understanding of all available options, and in particular with regard to the large number of supplementary health insurance policies. One participant noted a significant factor contributing to this difficulty is the lack of uniformity in policy names among health insurers. For instance, a policy named ‘conscious choice (bewuste keuze)’ may have different implications with health insurer X compared to health insurer Y.

Furthermore, several participants found the language used in insurance conditions to be overly complex or extensive, making it challenging for them to comprehend the content and its implications for them.

*‘Well*,* that is quite a task. You have to sit down and study it thoroughly*,* and the insurance language is not exactly the language that I am familiar with as a technician… And*,* in addition*,* in my situation*,* I find myself having to read things multiple times. By the end of the page*,* I can’t recall what was at the top of the page. So*,* I have to go back and read to see what was there because only then can I make a meaningful comparison’. (Participant #5)*

Finally, for more than half of the participants, the premium differences between the different health insurance policies are too small to delve into each of them annually. In general, the idea among participants is that it is not worth switching to another health insurance policy for a few euros of savings.

*‘I occasionally explore alternatives*,* but the differences in insurance policies are minimal. While there might be a potential saving of a few euros*,* it’s not significant enough for me to consider switching’. (Participant #1)*

4. What support do citizens want when choosing a policy

A number of participants indicated a preference for a return to the previous health insurance system in which a large number of insured did not have to make choices about a health insurance policy. Instead, they were simply covered through the national health insurance fund. Others, in addition, indicated that reducing the number of insurance policy options would be beneficial when choosing a health insurance policy.

*‘They shouldn’t make it so complicated*,* with all kinds of different types and forms of health insurance policies. I recall reading or seeing last year that there are 70 or 80 different ways to insure yourself. How do you make a choice from that’? (Participant #9)*

One participant suggested implementing a questionnaire for citizens that presents, instead of all policies, a manageable number of recommendations for policies aligning with their needs and preferences.

*‘I might be helped by filling in a questionnaire from which a distillation of policies would emerge. It would be beneficial to receive recommendations on the most suitable insurance for me*,* derived from answering around 30 questions…. I would definitely use that*,* if only to verify whether I made the right choice.’ (Participant #3)*

Furthermore, other participants mentioned that the provision of information about health insurance policies should be improved, and that they would benefit from a central and accessible information point where they can physically go to if they need help with their health insurance policy.

*‘I believe the information should be more transparent and straightforward. It would be beneficial to have a clear understanding beforehand*,* rather than being confronted with complexities afterwards. Just concrete*,* simple*,* and very clear information*,* which can be understood by everyone. Not a whole story with multiple lines*,* that’s not interesting. People don’t read that’. (Participant #16)*

*‘It would be beneficial to have a centralized point where one can readily go to and ask questions about what is covered or not. In the past, there used to be offices that served this purpose, but they are no longer in existence. It doesn’t necessarily need to be available all week, even a few hours per week would be sufficient.’ (Participant #7)*

Some participants indicated that they did not necessarily need help in choosing a health insurance policy themselves, but recognized the potential necessity for citizens who, for instance, face challenges in language proficiency or digital skills. The government and health insurers are seen as the most important parties to provide or coordinate this support by most participants. However, some participants also noted that citizens themselves should take more responsibility to familiarize themselves with health insurance policies.

## Discussion

This study explored what barriers citizens in the Netherlands with low HIL face during the selection process of a health insurance policy, and what their specific needs and preferences are regarding information and support. It can be concluded that not all citizens with low HIL, who were interviewed in this study, were sufficiently motivated to actively look for alternative health insurance options every year. This lack of motivation is partly due to the feeling that it is a task imposed upon them by the government, and because choosing a health insurance policy is perceived as too complicated and not sufficiently worthwhile. According to citizens with low HIL, the government and health insurers should strive to simplify the process of selecting a health insurance policy. This could be achieved, according the citizens with low HIL, by minimizing the number of health insurance options, enhancing the availability of information regarding health insurance policies, and ensuring easier access to this information.

It is important to consider the extent to which these findings are specific to citizens with low HIL. It is likely that there is also a lack of motivation among certain citizens with a high level of HIL when it comes to looking for health insurance policies, or, for example, among those with a substantial income who can relatively easy cover unforeseen healthcare costs. Moreover, as previously described in the “Introduction” section, for many citizens in the Netherlands, whether they have a low or a high level of HIL, the task of identifying a suitable policy among a wide range of policies and insurance options is difficult and time consuming [12–14]. We would like to emphasize that some recommendations in this study, for instance to develop targeted aid in helping to support citizens’ decision making when navigating health insurance policies, are not solely targeted at citizens with low HIL, but are likely extend to a broader group in the Netherlands. Other recommendations, for instance to develop health insurance information in non-digital formats, will particularly benefit citizens with low HIL. Finally, it is important to note our awareness that certain citizens, regardless of whether they have a high, or low, level of HIL, may not require assistance in selecting a health insurance policy.

The effective and transparent provision of information, tailored to the needs of citizens, is essential when navigating health insurance policy options [4]. There is a lot of, mostly digital, information available, and despite numerous initiatives, such as the “Health Insurance Card” (de Zorgverzekeringskaart) - aimed at providing comprehensive information to citizens about health insurances - several studies suggest that many individuals struggle to find relevant information during the process of selecting a health insurance policy [4, 32]. In line with these findings, our results show that many citizens with low HIL perceive choosing a health insurance policy as a difficult task, and several express a desire for improved support in this regard. This highlights the necessity for targeted decision-making aids to help empower citizens to make well-informed choices regarding their health insurance policies. An example of such a targeted decision-making aid is ‘Show Me My Health Plans’ SMMHP, developed in the US [33]. This programme is designed to provide education, an annual out of pocket cost calculator, and personalized plan recommendations. The results of SMMHP are promising, resulting in improved knowledge about health insurance, increased confidence in health insurance choices, and better HIL [33]. Introducing similar decision-making aids in the Netherlands could be useful for supporting citizens when navigating health insurance policies. With the help of such a tool to help with decision making, citizens would be shown fewer insurance options, potentially leading them to perceive the process of switching as less of a task.

Providing clear and practical information, through initiatives such as the SMMHP, is a crucial aspect of offering appropriate support to citizens in the process of selecting a health insurance policy. However, as outlined in the introduction, different types of skills are required to make health-related decisions. It is equally important to consider behavioural and affective attributes, emphasizing the ‘capacity to act’ [34]. Previous research highlights that many citizens are not well informed about the terms of their policies [16]. This finding is in line with our results showing that not all citizens with a low level of HIL are sufficiently motivated to actively delve into their health insurance options. We believe it is important to alert citizens to the fact that policy conditions change annually, and without proper awareness of these changes, they may encounter unexpected financial difficulties. Furthermore, it is crucial to identify which groups of citizens require extra assistance in the process of selecting a health insurance policy. Merely providing clear and practical information about health insurance, or raising awareness about the consequences of refraining from exploring health insurance options, will not support citizens who, for example, lack self-efficacy or possess insufficient digital skills. This group of citizens may require more personal attention when navigating the complexities of choosing a health insurance policy. For example, health insurance information in non-digital formats should be developed too. Follow-up research could focus on how to set this up properly.

Our findings show that most citizens with low HIL indicate that they are overwhelmed by the number of health insurance options and that they prefer fewer policies. Several reports suggest reducing the number of health insurance options or enhancing more transparency in the distinction between policies [9, 15, 35]. In this way, the overview of health insurance policies should become more clear and understandable for citizens, so that they will be more inclined to explore alternatives [5]. However, this trend has not been observed so far. The number of basic health insurance policies has remained consistent over the past five years, ranging between 55 and 60 different policies [23]. It appears unappealing for health insurers to offer fewer policies. Thus, without government intervention to change the law, it is unlikely that the number of health insurance policies will decrease in the coming years.

As previously mentioned, since the implementation of the Health Insurance Act in 2006, citizens in the Netherlands are assumed to play a more active role in the health insurance system than before. Our results show that among citizens with low HIL, there is resistance to delve into health insurance policies. Opponents of the current health insurance system notably stress that healthcare was previously more accessible and less complicated for citizens [36, 37]. Furthermore, trust in health insurers among citizens in the Netherlands is generally low, and there are many misconceptions about the different tasks of a health insurer [38]. As a result, overcoming resistance to the selection of health insurance policies appears to be a challenging task.

### Strengths and limitations

Little research has been done into the barriers faced by citizens in the Netherlands when choosing a health insurance policy. Our research aims to reduce this gap. In addition, a notable strength of this study is that, using the Nivel DHCCP, we were able to approach specifically those citizens with low HIL for an interview. Significantly they are the most vulnerable group when it comes to choosing and using a health insurance policy. A limitation of the study is that the participants were relatively old and highly educated. We were, partly because the sample consisted of only a handful of panel members who had a low level of education, unable to schedule an interview with one of this subgroup of citizens. As a result, no input was retrieved from these. In addition, there may also have been a non-response bias. Citizens who lack sufficient reading and writing skills, or do not have sufficient language proficiency, cannot participate in the Nivel DHCCP, and consequently, are excluded from research within the panel. It is likely that these citizens experience even more barriers that those who participated in the study. By providing participants with the option of conducting the interview by telephone, we were able to accommodate citizens who have poor digital skills. Finally, the findings of our study may not be fully applicable to other countries with choice-based health insurance system and should, therefore, be interpreted within the context of the specific healthcare system.

## Conclusion

Not all citizens in the Netherlands with a low level of HIL are sufficiently motivated to actively look for alternative health insurance options every year. This lack of motivation is partly due to their feeling that it is a task imposed upon them by the government, and because choosing a health insurance policy is perceived as too complicated and not sufficiently worthwhile. There is a need among citizens with a low level of HIL for clear and practical information about health insurance policies, especially from the government and health insurers. In addition to this need, we recommend enhancing an awareness of the consequences of neglecting to explore health insurance options. Moreover, more personal attention for the process of selecting a health insurance policy should be offered to vulnerable groups such as those who possess insufficient digital skills.

## Supplementary Information

Supplementary Material 1.

## Data Availability

The minimal anonymized data set is available upon request from prof. Judith de Jong (j.dejong@nivel.nl), project leader of the Dutch Health Care Consumer Panel, or the secretary if this panel (consumentenpanel@nivel.nl). The Dutch Health Care Panel had a program committee, which supervises processing the data of the Dutch Health Care Consumer Panel and decides about the use of the data. This program committee consists of representatives of the Dutch Ministry of Health, Welfare and Sport, the Health Care Inspectorate, Zorgverzekeraars Nederland (Association of Health Care Insurers in the Netherlands), the National Health Care Institute, the Federation of Patients and Consumer Organisations in the Netherlands, the Dutch Healthcare Authority and the Dutch Consumers Association. All research conducted within the Consumer Panel has to be approved by this program committee. The committee assesses whether a specific research fits within the aim of the Consumer Panel, which is to strengthen the position of the health care user.
