# Supplementary material for: How do citizens with low health insurance literacy choose a health insurance policy in the Netherlands? An interview study
Source: BMC Health Serv Res. 2024 Dec 18;24:1567. doi: 10.1186/s12913-024-12062-0 (PMC11654150; doi:10.1186/s12913-024-12062-0)
Supplement: Supplementary file 1 — Supplementary Material 1. [file 12913_2024_12062_MOESM1_ESM.docx]

**Supplementary File 1: Interview topic list**

Introductory questions

1. What kind of health insurance policy do you currently have?
2. Have you ever switched to another health insurance policy?

Main questions

1. To what extent and why do you consider choosing a health insurance policy:

- important?

- interesting?

- difficult?

1. What steps do you take when choosing a health insurance policy?
2. What barriers do you experience when choosing a health insurance policy?
3. How, and by whom, would you like to be supported in choosing a health insurance policy?
